# A pilot study of short T2* measurements with ultrashort echo time imaging at 0.35 T

**DOI:** 10.1186/s12938-018-0505-5

**Published:** 2018-06-04

**Authors:** Xiuyuan Chen, Bensheng Qiu

**Affiliations:** Center for Biomedical Engineering, University of Science and Technology of China, Jinzhai Road, NO.96, Hefei, 230026 China

**Keywords:** Low field MRI, UTE pulse sequence, T2* measurement

## Abstract

**Purpose:**

Ultrashort echo time (UTE) sequences play a key role in imaging and quantifying short T2 species. However, almost all of the relevant studies was conducted at relatively high fields. The purpose of this work was to further explore the feasibility of UTE imaging and T2* measurement for short T2 species at low fields.

**Methods:**

A 2D UTE sequence with an echo time (TE) of 0.37 ms was developed on a 0.35 T permanent magnet scanner. This sequence acquires multiecho images to fit the monoexponential signal decay model for quantitative T2* calculations. In the phantom experiments, MnCl_2_ solutions with different T2* values were used to assess the curve fitting model in low fields. In the in vivo experiments, T2* measurements were performed on the Achilles tendon of five normal volunteers.

**Results:**

The phantom studies showed a significant linear relationship between the MnCl_2_ solution concentration and R2* (1/T2*) values, which indicated the stability and accuracy of the T2* quantification model. The in vivo studies demonstrated that mean T2* value of Achilles tendon is 1.83 ± 0.21 ms, and the mean coefficient of determination (R-squared) was 0.996.

**Conclusions:**

Both phantom and in vivo experiments showed that UTE imaging and quantification for short T2 components were feasible at low field 0.35 T scanner. This pilot study presents preliminary conclusions for future work.

## Background

Biological tissues frequently contain different water components, which results in different distinct transverse relaxation times (T2) or apparent transverse relaxation times (T2*) during MR examination [1]. Quantitative relaxation time calculations are usually based on exponential fitting of data obtained from multiecho gradient echo sequences or Carr–Purcell–Meiboom–Gill sequences [2, 3]. However, there are a variety of short T2 tissues in the musculoskeletal system, and the average T2* values for these tissues range from several milliseconds down to tens of microseconds [4, 5]. Conventional magnetic resonance imaging employs T1 and T2 weighted sequences has a relatively longer echo time (TE). Even with gradient echo (GRE) sequences with TEs down to about 2 ms, short T2 tissues have little signal that can be detected [6].

Ultrashort echo time (UTE) technique using radial ramp sampling and half pulse excitation can achieve a minimal TE before the transverse relaxation signal has significantly decayed. Consequently, short T2 species that are invisible in conventional MRI can be imaged by UTE sequences [7, 8]. Multiecho images obtained from UTE sequences at varying TEs can be used to fit the exponential decay model for short T2* measurements. Biochemical changes in human tissues may affect the T2* values and thus can be detected and quantified with UTE sequences [9]. Therefore, researchers are paying more attention to quantifying short T2* calculations as an objective measure of tissue properties and health [10–14].

Previous studies on UTE imaging for short T2 tissues were focused on high fields, such as 1.5 T, 3.0 T [11–18] and higher [10, 19]. High field MR devices have available signal scales with B_0_^2^. However, the superconducting magnets required to generate B_0_ fields above 0.5 T are a major cost of a typical MRI installation. The continuing interest in low field MRI is stimulated by potential reductions in equipment and maintenance costs [20]. Therefore, low field scanners are still widely used in the clinic. It is of practical meaning for UTE sequences to quantitate short T2 species in low fields. On the one hand, low field MRI is inferior to high field MRI in terms of field homogeneity and signal-to-noise ratio (SNR), which may influence image quality and result in quantitation deviation. To ascertain the accuracy and robustness of short T2* measurements in low fields, we present a method, which employs the linear correlation between MnCl_2_ solution concentrations and R2* (1/T2*) values, to assess the quantitative model.

In this pilot study, a typical 2D UTE sequence was developed at 0.35 T permanent magnet scanner. Both phantom (a series of MnCl_2_ solutions with different concentrations) and in vivo (Achilles tendon from 5 healthy volunteers) experiments were conducted to demonstrate the feasibility of short T2* measurements at low fields.

## Methods

### Pulse sequence

**Fig. 1:**
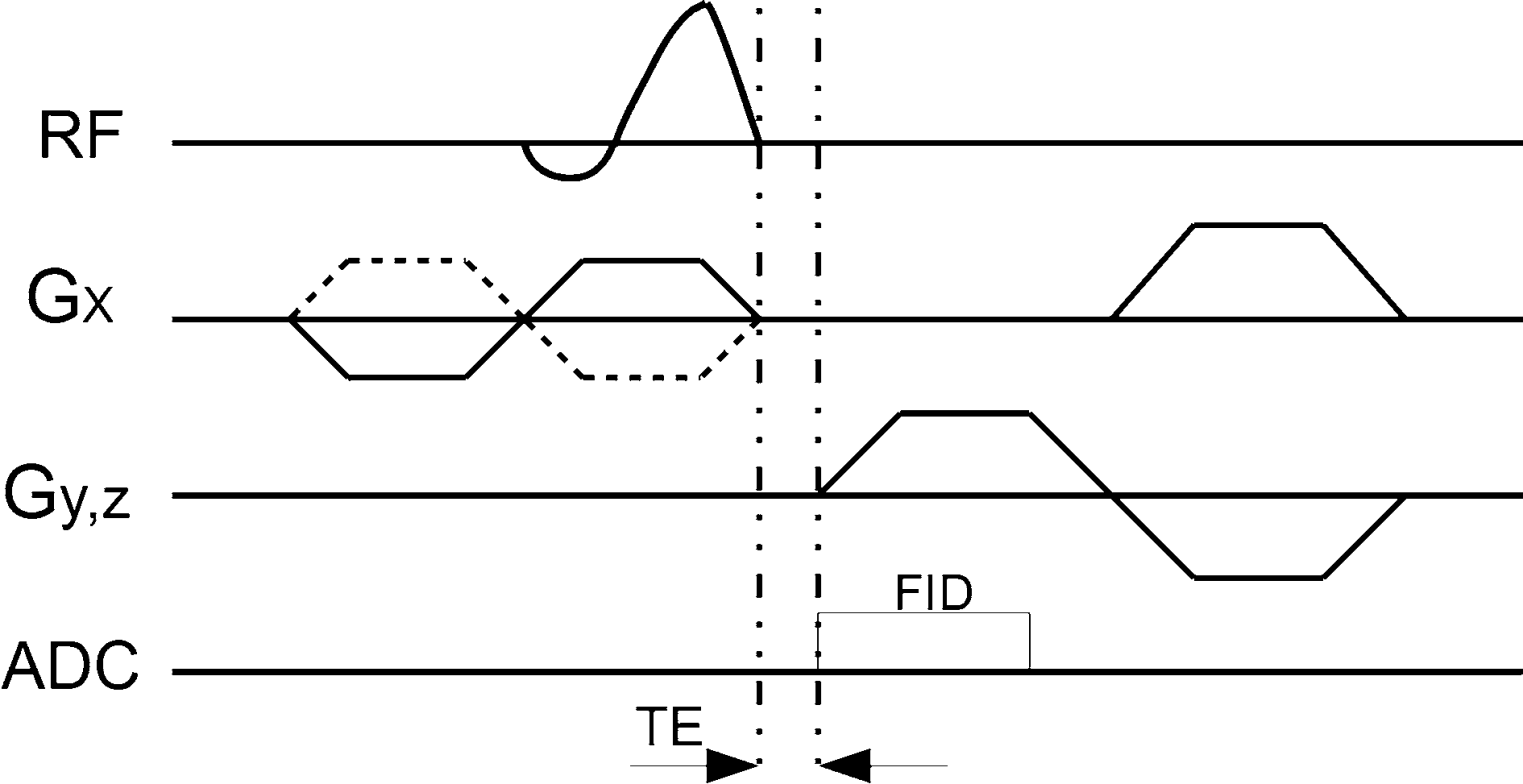
Diagram of a 2D UTE sequence. Radial ramp sampling, half-SLR RF and VERSE technique was used to achieve a short echo time. Duration time of RF transmitter turn-off is 20 μs, ADC duration before the readout gradient is 0.35 ms, and the total echo time is 0.37 ms

A 2D UTE pulse sequence was implemented on a 0.35 T MR system (PICA, Time Medical System, Taizhou, China) with a maximum gradient performance of 56 T/m/s and an amplitude of 28 mT/m. The pulse sequences used radial sampling and half-SLR radiofrequency (RF). The RF shape was redesigned by the variable-rate selective excitation (VERSE) technique to fit the down-ramp of the slice-select gradient. The sum of signals from two successive half RF pulse excitations with opposite polarity slice-select gradients composed a complete k-space line [11]. Data collection started before the readout gradient was ramped up in order to acquire the k-space center. Bipolar slice-select and readout gradients were used to reduce the eddy currents [12]. The minimum valid TE was only limited by the transmit/receive switching duration. A diagram of the UTE sequence is shown in Fig. 1. The actual TE can be as short as 0.37 ms.

### Phantom experiments

Manganese chloride (MnCl_2_) has been described as a contrast-variable MRI phantom [21]. In this study, highly concentrated MnCl_2_ solutions with short T2* values were used as a phantom. They were placed in four cylindrical plastic containers (diameter of 2 cm and volume of 50 ml) with concentrations of 16, 8, 4 and 2 mM, respectively. The sequence parameters were as follows: slice thickness = 5 mm, FOV = 200 mm, TR = 6 ms, flip angle = 90°, receiver bandwidth = 20,833 Hz, reconstruction matrix size = 256 × 256, 540 projections, 256 sampling points per projection, and 1.5 min per image. TEs of 0.5, 1, 1.5, 2, 2.5, 3, 3.5, 4 and 5 ms were used in the data acquisition. T2* values were calculated using the monoexponential attenuation model. The relation between T2* values and MnCl_2_ concentrations was explored.

### In vivo Achilles tendon experiments

Five healthy volunteers (4 female/1 male, mean age 24.8 ± 3.2 years, human ethics protocol Grant No. USTCEC201700006) were involved in this pilot study. Images were scanned by UTE sequence in the transverse plane. The signals from the ankles were collected by a knee receive coil. The sequence parameters were as follows: slice thickness = 5 mm, FOV = 200 mm, TR = 6 ms, flip angle = 90°, receiver bandwidth = 20,833 Hz, reconstruction matrix size = 256 × 256, 540 projections, 256 sampling points per projection, and 1.5 min per image. A conventional 2D spin echo (SE) sequence with a TE of 15 ms was used as a comparison. UTE sequences with TEs of 0.4, 0.6, 0.8, 1.4, 2.4 and 3.9 ms were performed for quantitative T2* calculation.

### Data processing

The data regridding technique was used in image reconstruction. The T2* value was measured by fitting a monoexponential attenuation model:

1 $$ S\left( {TE} \right) = S_{0} \times e^{{ - \frac{TE}{T2*}}} + {\text{N}} $$

where N was the background noise. The region-of-interest (ROI) was selected from each sample image and copied to the corresponding regions in images acquired at varying TEs. In the phantom experiments, the ROI contained 20 × 20 pixels in four MnCl_2_ solution regions. In the in vivo experiments, the manually selected ROI covered the whole Achilles tendon region. The signal Intensity within each ROI was used to fit the monoexponential model, and curve fitting was processed using the Curve Fitting Toolbox with the trust-region algorithm in MATLAB. The coefficient of determination (R-squared) was used for curve fitting quality evaluation. The relation between T2* values and MnCl_2_ solution concentrations was explored.

## Results

### Phantom experiments

**Fig. 2:**
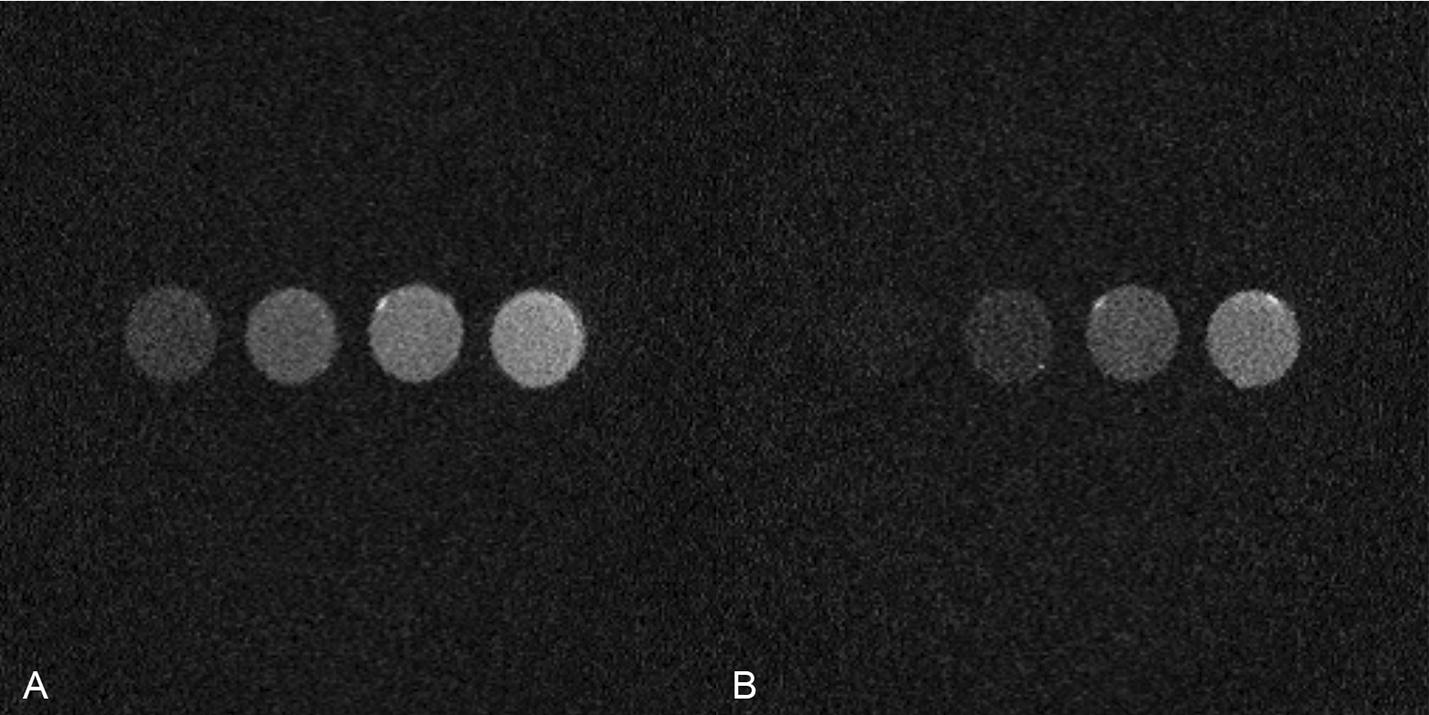
UTE imaging of MnCl_2_ solution at TE of 0.4 ms (**A**) and of 4 ms (**B**). The MnCl_2_ concentration is 16, 8, 4, 2 mM respectively from left to right

**Fig. 3:**
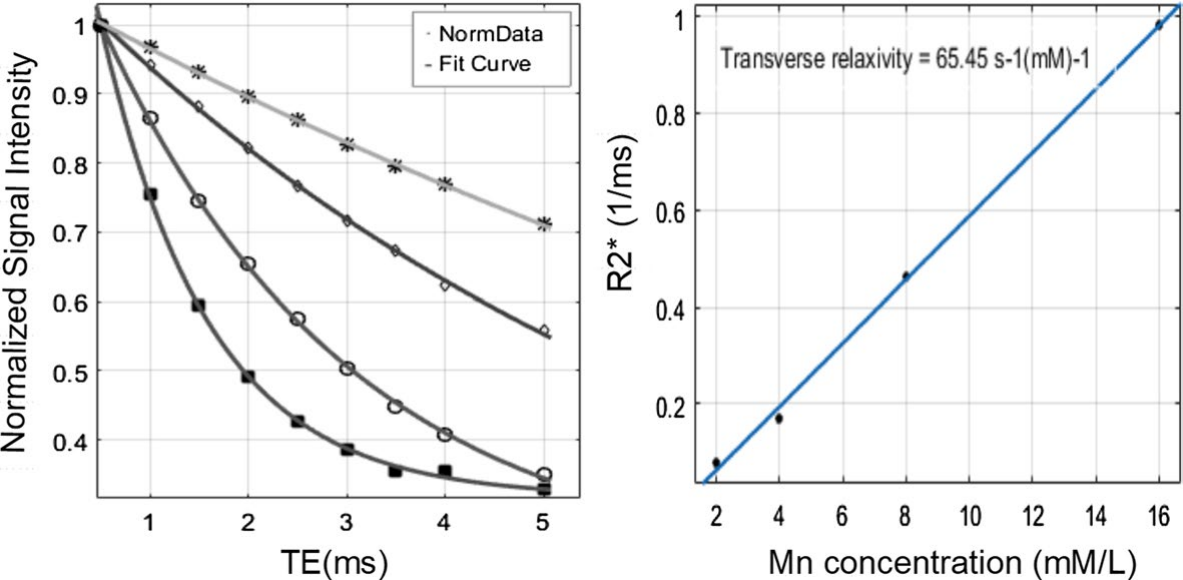
T2* decay curve of MnCl_2_ solution fitting the monoexponential model excellent. Decay velocity is inversely proportional to the Manganese concentration. Linear relationship between the manganese concentration and measured value of R2* (1/T2*) was clearly shown

The UTE images of MnCl_2_ solutions at TEs of 0.4 ms and of 4 ms are shown in Fig. 2. It is known that the higher the Mn concentration is, the smaller the T2 value is [22]. Hence, the images of different solutions had different signal intensities. In Fig. 2B, when the TE was 4 ms, shorter T2* solution signals were remarkably weak compared to those in Fig. 2A, when the TE was 0.4 ms. Figure 3 shows that the T2*s measured using the monoexponential attenuation model were 1.02 ms for 16 mM, 2.18 ms for 8 mM, 5.88 ms for 4 mM and 12.56 ms for 2 mM. The mean coefficient of determination (R-square) of model fitting was 0.9987. The second figure in Fig. 3 shows the linear relationship between R2* (1/T2*) values and MnCl_2_ concentrations. The relaxation rate was 65.5 s^−1^ mM^−1^, and the R-square of the linear fitting was 0.9983.

### In vivo Achilles tendon experiments

**Fig. 4:**
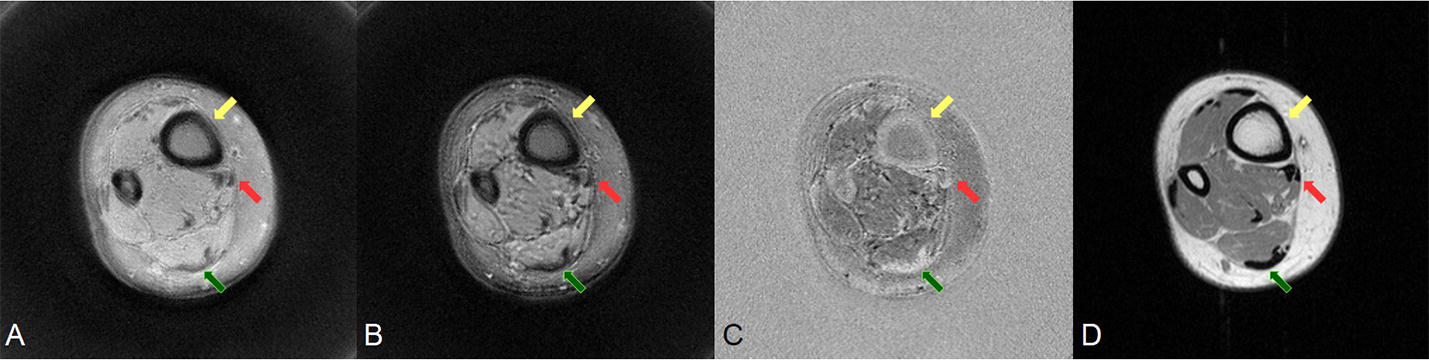
UTE images of ankle in axial with TE of 0.4 ms (**A**) and of 4 ms (**B**) was shown. This two echo subtraction image (**C**), Signal of Achilles tendon (green arrow) and muscle tendon (red arrow) was significant reduced in second image. In corresponding spin echo image (**D**), all the short T2 tissues have no signal

UTE images acquired at a TE of 0.4 ms (A) and a TE of 4 ms (B), subtraction images (C) and the corresponding T1 weighted SE images (D) are shown in Fig. 4. In the SE image, the Achilles tendon, muscle tendon and cortical bone are completely black. In the UTE images, the signal of those short T2 tissues can be detected. Subtract the second echo image (B) from the first echo image (A) can get the echo subtraction image (C). It is known that the decay rate of short T2 tissues is quicker than that of long T2 tissues, so the gap between two echo images of short T2 tissues is larger. Therefore, the brighter signal of tendons in Fig. 4C means that signal of tendons was significant reduced in Fig. 4B. Echo subtraction suppresses the long T2 muscle and fat signals, increasing the contrast with the tendons. However, the residual long-T2 signal limits the achievable contrast [4].

**Fig. 5:**
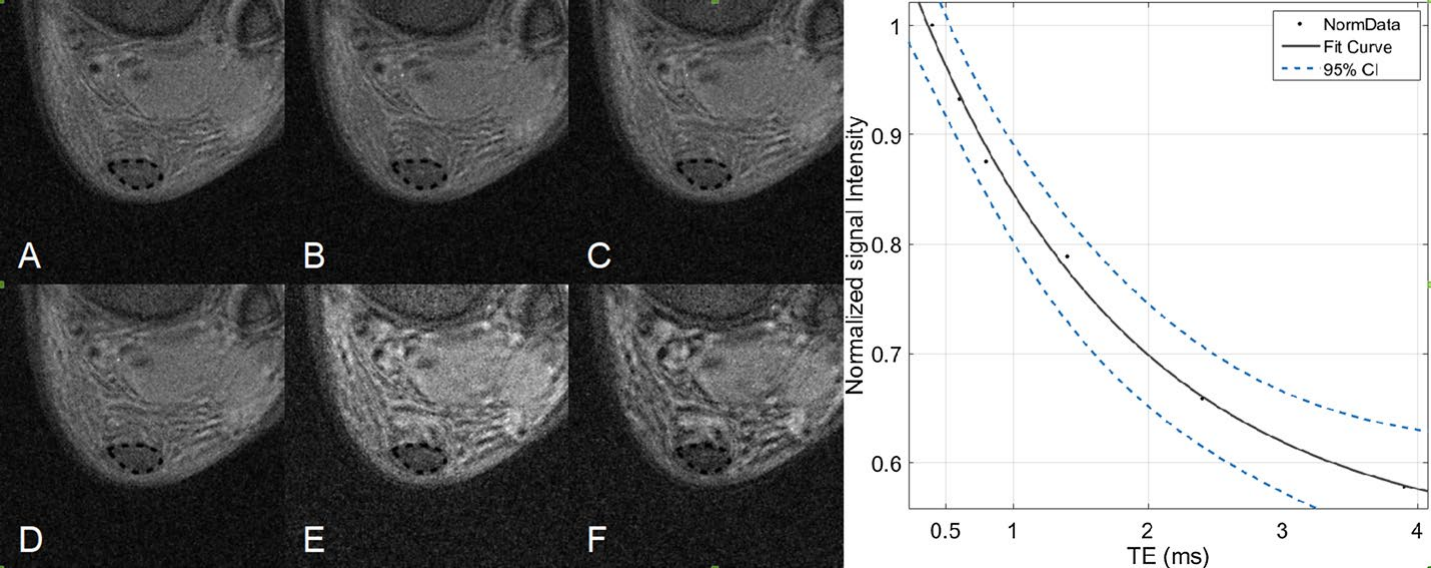
UTE images of Achilles tendon with TEs of 0.4, 0.6, 0.8, 1.4, 2.4 and 3.9 ms (**A**–**F**). Region of interest was marked with dotted lines. T2* decay curves of ROI in Achilles tendon was fitting the monoexponential model, 95% confidence interval for the fitting function was shown with dotted lines

Figure 5 shows the UTE images of healthy volunteer ankles at different TEs. The normalized signal intensity within the ROI was used for fitting the monoexponential decay model. The 95% confidence interval is shown by a dotted line. The experimental results are summarized in Table 1. T2*s ranged from 1.63 to 2.15 ms, the mean and standard deviation was 1.83 ± 0.21 ms, and the mean coefficient of determination (R-squared) was 0.996.

**Table 1:** T2* measurements of Achilles tendon in five healthy volunteers

Subject	1	2	3	4	5
T2* (ms)	1.913	1.695	1.786	2.15	1.63
R-square	0.9983	0.9958	0.993	0.9955	0.998
Mean ± SD (ms)	1.8348 ± 0.2058				

## Discussion and conclusion

The purpose of this study is to investigate short T2* measurement with UTE imaging in low field MRI. In general, T2 is not affected much by the main magnetic field strength [23, 24]. T2* is related to T2 by 1/T2* = 1/T2 + γ × Δ*B*, so it is sensitive to multiple factors, such as the magnetic susceptibility effect, field strength and homogeneity. There is evidence that T2* values increase as the field strength decreases, because the magnetic susceptibility effect is proportional to *B*_0_ [19, 25]. This means that lower fields have a potential advantage for short T2* measurements. However, field inhomogeneity in permanent magnets is a negative factor, and the lower SNR in low fields is also a problem for quantitative processes, especially when the short T2 species present a low signal at a relatively longer TE. Generally speaking, T2* value is vulnerable to MRI system changes. Quantitative UTE is a relatively novel technique to quantify short T2* species. For short T2*s, studies have found a linear relationship between the relaxation rate R2*(1/T2*) and the MnCl_2_ concentration [22, 26]. This relationship is not susceptible to device changes, and it can be used as a cross-platform reference to assess the precision of quantitation.

In this study, a set of phantom experiments was conducted and analyzed. The T2* value of the high concentration MnCl_2_ solution was selected to approximate that of short T2 tissues. Phantom images and monoexponential decay curve fitting achieved good performance. More importantly, there was strong and linear correlation between R2* and MnCl_2_ concentration, which indicated the accuracy and robustness of the short T2* measurements in low field MRI.

For in vivo experiments, oversampling and small receiver bandwidth were used to overcome the relatively low SNR at low fields. It is known that T2* varies with MR devices. In a study by Du et al. [18], a MnCl_2_ solution at 24 mM had a T2* value of 0.4 ms at 3 T, and the corresponding value in our work at 0.35T was 0.64 ms according to the linear fitting. The decreased T2* at higher field strengths is caused by magnetic susceptibility effects. According to previous reports at 3 T, T2* values of a large normal central region of the Achilles tendon in vitro ranged from 1.76 to 2.64 ms [16], in another study, the T2* of fresh in vitro Achilles tendon was 1.18 ± 0.45 ms [17]. In our work, the mean T2* value was 1.83 ± 0.21 ms. This result is within the normal range, considering the differences in magnetic field and specimens.

We have shown that quantitative UTE for short T2* species can be successfully implemented in low field MRI. However, there are several limitations to our study. First, our sample size was small, and more experiments on different tissue specimens will be carried out in future work. Second, T2* measurement by a monoexponential model is only an approximation of all the components. Bicomponent analysis or monocomponent analysis after suppressing the long T2 component signal, can provide the relaxation time of a single component [1, 12]. Third, the valid echo time in this study is not sufficient to image the cortical bone with a T2* value of approximately 0.4–0.5 ms [15]. Higher-performance hardware can be used to reduce the TE and improve the signal strength of short T2 tissues.

In summary, in this pilot study, phantom experiments indicated the stability and accuracy of the T2* quantification model. In healthy human subjects, the mean T2* value of Achilles tendon was 1.83 ± 0.21 ms. The feasibility of short T2* measurements with UTE sequences at 0.35 T was demonstrated. Future work will include an increased number of normal subjects and patients for clinical applications.
